# Genome-wide analysis and functional characterization of the DELLA gene family associated with stress tolerance in *B. napus*

**DOI:** 10.1186/s12870-021-03054-x

**Published:** 2021-06-22

**Authors:** Rehman Sarwar, Ting Jiang, Peng Ding, Yue Gao, Xiaoli Tan, Keming Zhu

**Affiliations:** Institute of Life Sciences, Jiangsu University, Zhenjiang, China

**Keywords:** *Brassica napus*, Gibberellins, DELLA, Abiotic stress, Genome-wide

## Abstract

**Background:**

*Brassica napus* is an essential crop for oil and livestock feed. Eventually, this crop's economic interest is at the most risk due to anthropogenic climate change. DELLA proteins constitute a significant repressor of plant growth to facilitate survival under constant stress conditions. DELLA proteins lack DNA binding domain but can interact with various transcription factors or transcription regulators of different hormonal families. Significant progress has been made on *Arabidopsis* and cereal plants. However, no comprehensive study regarding DELLA proteins has been delineated in rapeseed.

**Results:**

In our study, we have identified 10 *BnaDELLA* genes. All of the *BnaDELLA* genes are closely related to five *AtDELLA* genes, suggesting a relative function and structure. Gene duplication and synteny relationship among *Brassica. napus*, *Arabidopsis. thaliana*, *Brassica rapa*, *Brassica oleracea*, and *Brassica nigra* genomes were also predicted to provide valuable insights into the *BnaDELLA* gene family evolutionary characteristics. Chromosomal mapping revealed the uneven distribution of *BnaDELLA* genes on eight chromosomes, and site-specific selection assessment proposes *BnaDELLA* genes purifying selection. The motifs composition in all *BnaDELLA* genes is inconsistent; however, every *BnaDELLA* gene contains 12 highly conserved motifs, encoding DELLA and GRAS domains. The two known miRNAs (bna-miR6029 and bna-miR603) targets *BnaC07RGA* and *BnaA09GAI,* were also predicted. Furthermore, quantitative real-time PCR (qRT-PCR) analysis has exhibited the *BnaDELLA* genes diverse expression patterns in the root, mature-silique, leaf, flower, flower-bud, stem, shoot-apex, and seed. Additionally, *cis*-acting element prediction shows that all *BnaDELLA* genes contain light, stress, and hormone-responsive elements on their promoters. The gene ontology (GO) enrichment report indicated that the *BnaDELLA* gene family might regulate stress responses. Combine with transcriptomic data used in this study, we detected the distinct expression patterns of *BnaDELLA* genes under biotic and abiotic stresses.

**Conclusion:**

In this study, we investigate evolution feature, genomic structure, miRNAs targets, and expression pattern of the *BnaDELLA* gene family in *B. napus*, which enrich our understanding of *BnaDELLA* genes in *B. napus* and suggests modulating individual *BnaDELLA* expression is a promising way to intensify rapeseed stress tolerance and harvest index.

**Supplementary Information:**

The online version contains supplementary material available at 10.1186/s12870-021-03054-x.

## Background

Since the 1970s, *Brassica napus* has become the world's most economically valuable crop [1]. In recent years significant progress has been made in advancing *B. napus* selective breeding to remove undesirable components for high-quality vegetable oil and palatable livestock feed. Unfortunately, *B. napus* yield is susceptible to various abiotic and biotic stresses, such as higher salinity, drought, high/low temperature, and pathogen infections. These stresses have led to severe loss in harvest index and oil production in many regions of the world and limited its geographical distribution [2, 3]. Consequently, the effects of environmental stresses on *B. napus* cultivation eventually losing their economic importance.

Plants as sessile organisms evolved varied strategies to modulate their physiology to cope with fluctuating environmental conditions [4]. Tremendous work has been done to understand the role of plants biochemical, molecular, and cellular responses to abiotic and biotic stresses [5, 6]. These studies suggested that phytohormones are the critical components that convey the internal and external stimuli to facilitate plant adaptive response to environmental challenges. Among these hormones, Gibberellins (GAs) are considered one of the most vital phytohormones that dramatically affect plant physiology by crosstalking with multiple hormones [7, 8]. However, under external pressure, plants mediate GAs and other phytohormones homeostasis through a family of coregulators DELLA proteins to balance the growth in reserving resources for plant survival [9, 10]. A significant function of the DELLA proteins is to regulate the wide variety of transcriptional factors (TFs) and transcriptional regulators (TRs) of multiple phytohormones. For instance, DELLA proteins interact with transcriptional factors, including PHYTOCHROME INTERACTING FACTORs (PIFs), BRASSINOSTEROID INSENSITIVE 1 (BZR1), and EXPANSIN-A2 (EXP2), in a light-dependent and temperature-dependent manner to suppress cell elongation and cell proliferation [11–13], or interacting with DEHYDRATION-RESPONSIVE ELEMENT-BINDING PROTEIN 1B (DREB1B), JASMONATE ZIM-domain 1 (JAZ1), and TEOSINTE BRANCHED1/CYCLOIDEA/PCF (TCPs) to prime defense focusing on plant survival rather than its growth [14–16]. DELLA proteins are the sub-family of the transcriptional regulators GRAS (named after GIBBERLIC ACID INSENSITIVE 1, REPRESSOR OF GAI-3, and SCARECROW) [17]. Most GRAS subfamilies contain common N-terminal motifs, while DELLA proteins had a few α-helical segments called DELLA, LEXLE, and THYNP that have been termed the DELLA domain [18, 19]. Previously, it was proposed that mutation in 17 amino acids of the DELLA N-terminal region resulted in severe dwarf transgenic plant *gai-1* with dark green leaves but insensitive to salt and drought stress [20, 21]. Later it was demonstrated that the N-terminal region of the DELLA domain is responsible for *DELLAs* stability, which is operated by a GAs receptor Gibberellin insensitive Dwarf 1 (GID1) in GAs dependent and independent manner to foster plant growth by lifting *DELLAs* repression [22–24].

Rice, barley, tomato contain only one DELLA gene, *SLR1* (SLENDER RICE1), *SLN1* (SLENDER1), and *PROCERA,* respectively [9, 25, 26], while pea and maize hold two highly conserved *DELLA* genes. *LA*, *CRY,* and *d8*, *d9*, respectively [27, 28]. Additionally, researches on *Arabidopsis thaliana* reported the presence of five *AtDELLA* genes GA-Insensitive (*GAI*), Repressor of ga1-3 (*RGA*), RGA-Like1 (*RGL1*), (*RGL2*), (*RGL3*). Molecular cloning of the single and multiple *AtDELLA* genes in GA deficient mutant *ga-1* suggested the overlapping and unique roles of *AtDELLAs* in regulating GAs stimulated plant growth. For instance, *AtGAI* and *AtRGA* have been indicated as notable repressors of the plant vegetative growth [9, 29, 30], whereas *AtRGL1* and *AtRGL2* repressed floral augmentation and seed germination [31–34]. *AtRGL3* recently got attention in plant defense by positively regulating the jasmonic acid (JA), and salicylic acid (SA) mediated response against pathogen infections [10, 35].

Work over the last decade, the impact of *DELLAs* on seeded plant productivity has been progressively investigated. Apart from the exogenous splattering of the GAs to improve plant growth by repressing the repressor, one key factor was to alter GAs synthesis by fine-tuning the *DELLAs* activity for the amelioration of semi-dwarf varieties [26, 36, 37]. This results in enhanced plant tolerance to abiotic stresses, which ultimately improves crops harvest index and survival [38–40]. In addition to this, a recent study identifies a DELLA loss of function semi-dwarf mutant *ds-3* in oilseed rape, which confers a similar phenotype to previously reported semi-dwarf varieties, with resistance to lodging stress [41]. However, the *DELLA* gene family molecular mechanism and characterization in *B. napus* have not been well reported. Moreover, diversification of *DELLA* gene family during *B. napus* polyploidization would be of interest.

In this study, 10 members of the *BnaDELLA* genes were systematically characterized and analyzed by their phylogenetic and syntenic relationship, subcellular localization, protein motifs, gene structure, and *cis*-elements in the *B. napus* genome. Furthermore, expression profiles of the *BnaDELLAs* in eight different tissues, root, mature-silique, leaf, flower, flower-bud, stem, shoot-apex, seed, were analyzed using the qRT-PCR. Pre-published RNA-Seq data were also predicted to investigate *BnaDELLAs* expression patterns under different stress conditions such as cold, heat, drought, abscisic acid (ABA), salinity, and *Sclerotinia sclerotiorum* infection. Gene Ontology (GO) and miRNAs targeting the *BnaDELLA* gene family were also examined to characterize *BnaDELLAs* role. These results will provide valuable insights to illustrate the multiple functions of the DELLA proteins in *B. napus* and a basis for further genetic manipulation toward developing *B. napus* variants with increased stress tolerance to environmental fluctuation.

## Results

### Identification and characterization of *BnaDELLAs*

We have identified 10 *BnaDELLAs* in *B. napus* using the known five *A. thaliana DELLAs* (*GAI, RGA, RGL1, RGL2, RGL3*) peptide sequence as queries, and performed BLASTP searches in the *B. napus* genome database (GENOSCOPE http://www.genos cope.cns.fr/brass icanapus/) [42]. To confirm the BnaDELLA proteins integrity in the *B. napus*, we further analyze the retrieved sequences in different *B. napus* cultivar Zhongshuang 11 (ZS11) genome browser (BnPIR, http://cbi.hzau.edu.cn/bnapus), and manually corrected the redundant sequence information of the *BnaDELLAs* and named them according to their loci. Based on these methods, we found that each member in the *AtDELLA* gene family corresponds to multiple homologs in the *B. napus* genome (Table 1). Simultaneously, five *DELLA* genes in *B. rapa*, four in *B. oleracea,* nine *in B. juncea,* and five in *B. nigra* were classified using the same methods. We found that 10 *BnaDELLAs* members are derived from their progenitor *B. rapa* and *B. oleracea*. The genomic sequence length of the *BnaDELLAs* ranged from 1524-1740 bp, with a molecular weight varying from 55.83 to 63.32 KD (Table 1). Moreover, the isoelectric point (pI) values of the BnaDELLA proteins ranged from 4.69 to 5.94, which shows that these proteins are highly acidic. Besides, all BnaDELLA proteins showed a negative value of the grand average of hydrophobicity (GRAVY), indicating that BnaDELLAs are hydrophilic proteins. Moreover, the 10 BnaDELLA proteins subcellular localization signals were detected in the nucleus, which exhibits their transcriptional regulator role. The names of the *BnaDELLAs* and their locus id are also shown in (Table 1).

**Table 1:** Characterization of BnaDELLA family proteins

Group	Gene Name	Gene LOCUS ID	Chromosome Number	Location			Protein							Arabidopsis Orthologs		Subcellular Location
				Start	End	Orientation	ORF	AA	PI	Mw (Da)	GRAVY		AI			
I	*BnaA09GAI*	*BnaA09G0218400ZS*	scaffoldA09	15,333,875	15,336,195	Reverse	1740	579	5.23	63,323.35	-0.247	81.07			*AT1G14920 (AtGAI)*	Nucleus
	*BnaC09GAI*	*BnaC09G0254100ZS*	scaffoldC09	23,759,020	23,761,368	Reverse	1710	569	5.32	62,327.38	-0.232	82.65				Nucleus
	*BnaA06RGA*	*BnaA06G0409200ZS*	scaffoldA06	46,345,733	46,347,952	Forward	1722	573	5.58	62,511.61	-0.203	82.25			*AT2G01570 (AtRGA)*	Nucleus
	*BnaC07RGA*	*BnaC07G0269400ZS*	scaffoldC07	41,375,597	41,377,853	Reverse	1734	577	5.41	62,782.98	-0.177	83.19				Nucleus
II	*BnaC02RGL1*	*BnaC02G0205300ZS*	scaffoldC02	17,476,351	17,477,874	Forward	1524	507	5.37	55,827.07	-0.159	92.92			*AT1G66350 (AtRGL1)*	Nucleus
	*BnaA02RGL1*	*BnaA02G0160500ZS*	scaffoldA02	9,357,771	9,367,517	Forward	1527	508	5.94	56,584.09	-0.215	89.84				Nucleus
III	*BnaA05RGL2*	*BnaA05G0486300ZS*	scaffoldA05	44,153,415	44,155,599	Forward	1635	544	4.71	59,184.6	-0.189	85.86			*AT3G03450 (AtRGL2)*	Nucleus
	*BnaA05RGL2-2*	*BnaA05G0485400ZS*	scaffoldA05	44,093,973	44,096,162	Forward	1641	546	4.69	59,456.9	-0.188	86.08				Nucleus
	*BnaC09RGL3*	*BnaC09G0489900ZS*	scaffoldC09	60,072,165	60,073,742	Forward	1578	525	4.8	57,604.95	-0.211	88.8			*AT5G17490 (AtRGL3)*	Nucleus
	*BnaA10RGL3*	*BnaA10G0194400ZS*	scaffoldA10	21,533,732	21,535,309	Forward	1578	525	4.78	57,634.01	-0.203	89.16				Nucleus

*ORF* (Open reading frame); *AI* (Aliphatic index); *pI* (Isoelectric point); *GRAVY* (Grand average of hydropathicity)

### Evolutionary relationship and gene structure analysis of *BnaDELLAs*

**Fig. 1:**
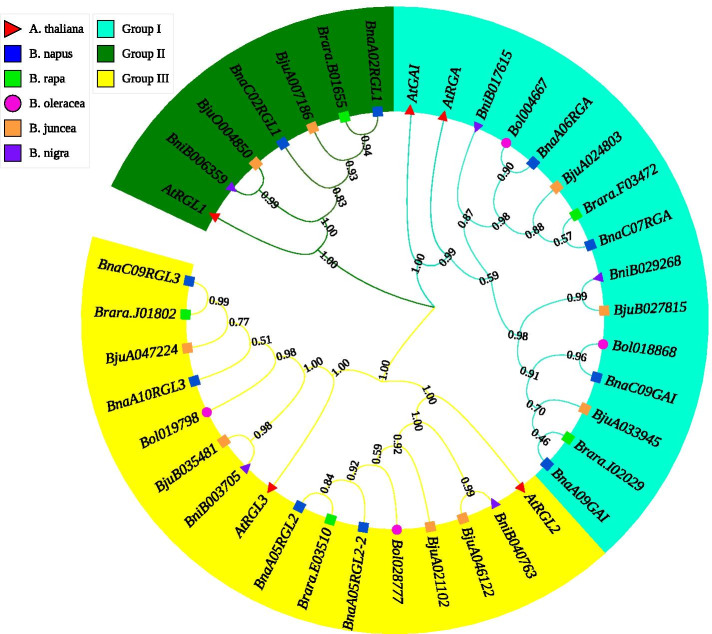
The cladogram of DELLA proteins from *A. thaliana* (At:5), *B. napus* (Bna: 10), *B. rapa* (Bra: 5), *B. oleracea* (Bol: 4), *B. juncea* (Bju: 9), and *B. nigra* (Bni: 4) were conducted in MEGA X [86] using the neighbor-joining method, missing data with gaps were eliminated by complete deletion option. The DELLA proteins are cluster into three groups, which are indicated by the different colors. The bootstrap test (1000 replicates) is shown next to the branches

**Fig. 2:**
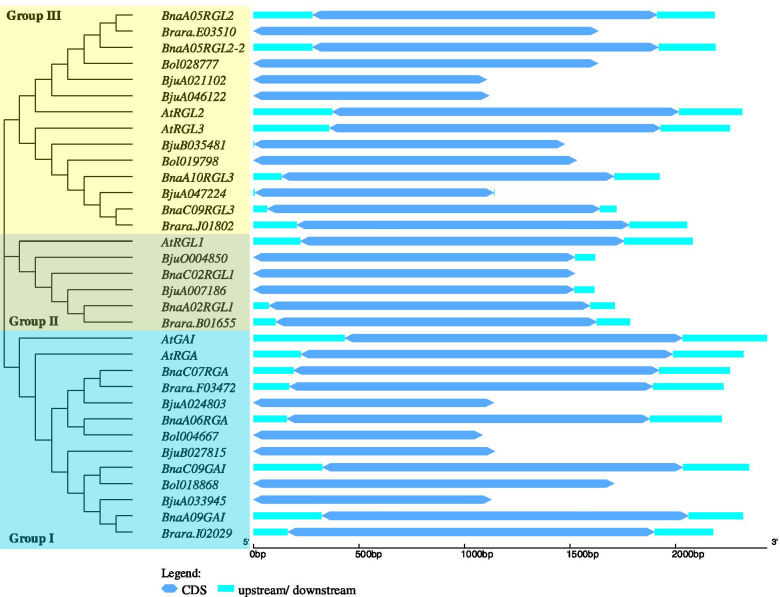
Exon and intron location of the *BnaDELLAs*. Blue double-sided wedge represents exon, and upstream/downstream regions are indicated as cyan-colored boxes. The scale can estimate the length of the exon at base

The *DELLAs* evolutionary history among six *Brassicaceae* species *A. thaliana* (At), *B. napus* (Bna), *B. rapa* (Bra), *B. oleracea* (Bol), *B. juncea* (Bju)*,* and *B. nigra* (Bni) was deduced using the neighbor-joining method. Based on the phylogenetic analysis, 38 *DELLA* genes in which five *AtDELLAs*, 10 *BnaDELLAs*, five *BraDELLAs*, four *BolDELLAs*, nine *BjuDELLAs,* and five *BniDELLAs* were cluster into three groups according to the topologies and bootstrap support (Fig. 1). Group I contain GAI and RGA clade, Group II holds RGL1 clade, Group III holds RGL2 and RGL3 clade. *B. napus DELLA* genes were relatively closer to the *A. thaliana*. However, *B. napus* and *B. rapa DELLAs* show 100% similarity between each other. Besides, a homolog of *AtRGL1* was not identified in the *B. oleracea* compared to those of *B. napus, B. rapa*, *B. juncea,* and *B. nigra.* This might be due to gene loss during the evolution process or the emerging genome gaps in *B. oleracea*. However, in *B. napus*, Group I, II, III had four, two, four *DELLA* members, respectively. *DELLA* genes grouped into the same subfamily are previously known to have distinct or overlapping functions [21, 31, 35, 43]. To recognize the *DELLA* genes family diversification in *B. napus*, we have implemented the Gene Structure Display (GSDS) web analysis by comparing the coding sequence (CDS) and corresponding genomic sequences of *AtDELLAs*, *BnaDELLAs*, *BraDELLAs*, *BolDELLAs,* and *BjuDELLAs.* As shown in Fig. 2, members of the *DELLA* genes among denoted species are highly conserved and intron-less with only one exon. Moreover, the exon location of *DELLAs* among different phylogenetic-related species is conserved, suggesting a similar evolutionary relationship. However, the length of exon among the *DELLA* subfamily was different. For example, *BnaRGL1* exon length was smaller than other *BnaDELLAs* members in Group I and Group III, indicating gene structure diversification. In summary, the gene structure of the *DELLA* genes from different *Brassicaceae* species is highly conserved, with some difference in the exon length (Fig. 2).

### Multiple sequence alignment and analysis of *BnaDELLAs* motifs

The putative sequences of the DELLA proteins from *B. napus, A. thaliana, B. rapa, B. oleracea, B. juncea,* and *B. nigra* were aligned to explore amino acid conservation in *B. napus*. Based on the alignment, we found five homologs DELLA proteins from *A. thaliana* show higher percent amino acid similarity with *B. napus* (Table S1). Similar to *A. thaliana*, the *B. napus* and other denoted species contain highly conserved DELLA and GRAS domains at the N-terminal and C-terminal region, respectively. It was known that the N-terminal DELLA domain is involved in stabilizing the *DELLA* gene activity [18, 44], while the GRAS domain acts as a coregulator to interact with several transcriptional factors and regulators (Figure S1) [37, 45, 46]. The presence of the (VHIID-PFYRE-SAW) and two leucine heptad repeats LHRI and LHRII on the C-terminal of the GRAS domain are responsible for the protein interaction [47, 48]. However, some studies have also proposed DELLA domain lower-affinity with intrinsically unstructured proteins [45, 49]. Overall, the domain arrangements in the *B. napus DELLA* gene family are comparable to *A. thaliana, B. rapa, B. oleracea, B. juncea,* and *B. nigra*. The secondary structure feature (alpha-helix and Beta sheets) from the *AtRGL1* accessions number (*At1G66350.1*) was displayed in Figure S1. However, the predicted secondary structures of all *DELLA* genes from the denoted plant species were relatively different.

**Fig. 3:**
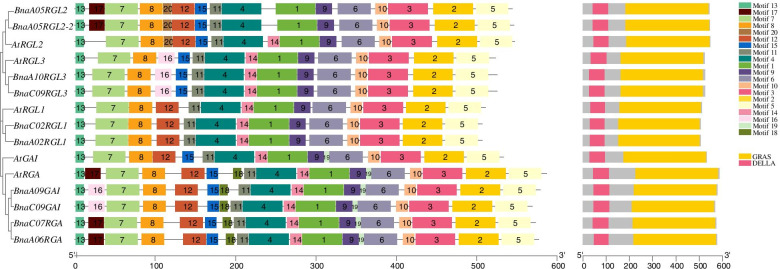
The length of the 20 motifs ranged from 6 to 50 amino acid residues and represented by different colors and numbers, *p- values* of the motifs on each protein is less than 1e^−5^

To gain more insights into the diversity of *BnaDELLAs* in *B. napus,* we generated a graph showing domains and their position on AtDELLAs and BnaDELLA protein members. We found that the DELLA and GRAS domains are conserved in all DELLA proteins of *A thaliana* and *B. napus*, but motifs were unevenly distributed (Fig. 3). Every BnaDELLA member contains four to 16 conserved motifs, and their length ranged from six to 50 amino acids. Motif 1 to 13 were identified in all groups except *AtRGL3, BnaA10RGL3,* and *BnaC09RGL3* lacking motif 12. In which motif 7 and 8 are annotated as DELLA domain (Fig. 3). Moreover, Motif 14 and 15 were not detected in *AtRGL2*, *BnaA05RGL2*, *BnaA05RGL2-2,* and Group II, respectively. Motif 16 was detected in *AtRGL3, BnaA10RGL3*, *BnaC09RGL3, BnaA09GAI*, and *BnaC09GAI*. Furthermore, Motif 17 was only present in the N-terminal region of the *AtRGA, BnaC07RGA, BnaA06RGA, BnaA05RGL2*, and *BnaA05RGL2-2* genes*.* Motif 18 was detected in *AtRGA*, *BnaA09GAI*, *BnaC09GAI, BnaC07RGA,* and *BnaA06RGA.* In contrast, Group I, *AtRGL2*, *BnaA05RGL2*, and *BnaA05RGL2-2* had an extra motif 19 and 20, respectively. These results exhibit that the *BnaDELLAs* subfamilies differ in motif arrangements, indicating the *BnaDELLA* gene family functional divergence during duplication events. However, proteins with similar motifs arrangements specified the functional similarities among *BnaDELLA* members. A schematic logo diagram of *BnaDELLAs* motifs was shown in Figure S2.

### Chromosome location and collinearity analysis

**Fig. 4:**
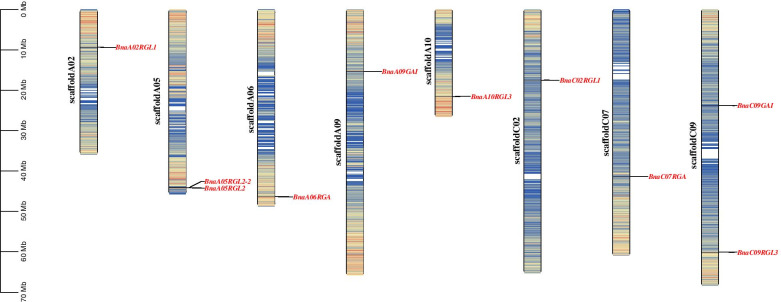
Schematic representation of DELLA genes distribution on *B. napus* chromosomes. Chromosome number is indicated on the side of each chromosome

**Fig. 5:**
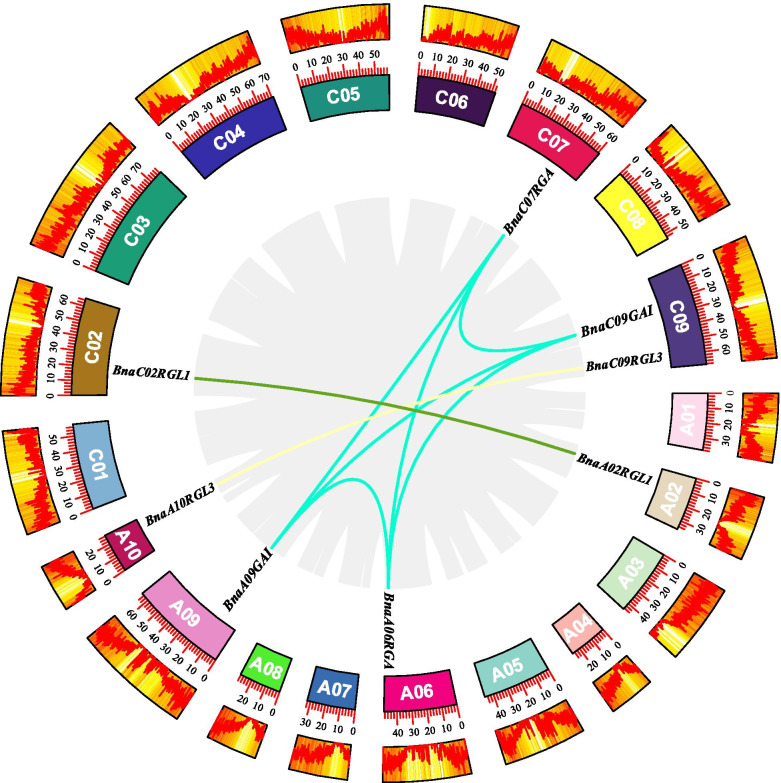
Synteny analysis of the *BnaDELLA* family in *B. napus*. Cyan-colored line genes belong to Group I, and green-colored line genes belong to group II, yellow-colored line genes belong to Group III. These colored genes lines indicate duplicated *BnaDELLA* gene pairs, while gray lines in the background represent synteny blocks in the *B. napus* genome. The distribution density of *BnaDELLAs* present at the bottom of each chromosome

**Fig. 6:**
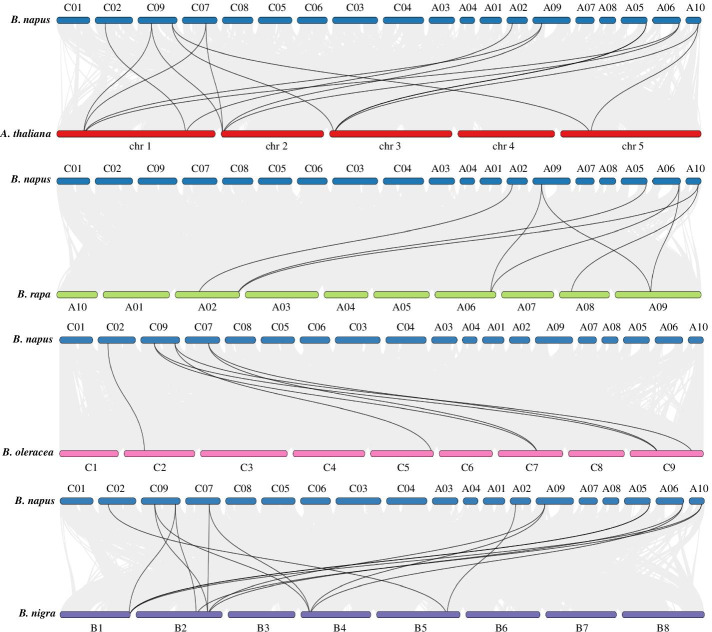
Synteny analysis of *BnaDELLAs* between *A. thaliana, B. rapa, B. oleracea,* and *B. nigra.* Black lines indicate the syntenic *DELLA* gene pairs between denoted species. While in the background, gray line represents collinear blocks

Chromosomal mapping analysis showed that 10 *BnaDELLAs* distributed on eight *B. napus* scaffolds (Fig. 4), which have not been assembled into a chromosome. Furthermore, no distribution of *BnaDELLAs* were observed in the scaffoldA01, scaffoldA03, scaffoldA04, scaffoldA07, scaffoldA08, scaffoldC01, scaffoldC03, scaffoldC04, scaffoldC05, scaffoldC06, and scaffoldC08. However, six *BnaDELLAs*, including, *BnaA02RGL1*, *BnaA05RGL2, BnaA05RGL2-2*, *BnaA06RGA*, *BnaA09GAI*, and *BnaA10RGL3*, are located on the AA subgenome. In contrast, four *BnaDELLAs,* including *BnaCO2RGL1*, *BnaCO9GAI*, *BnaC07RGA,* and *BnaC07RGL3*, located on the CC subgenome, suggesting the uneven distribution of *BnaDELLAs* in the *B. napus* genome (Fig. 4). Furthermore, by using the BLAST and MCScanX methods, we detected the six segmental duplication pairs such as *BnaA09GAI/BnaC09GAI*, *BnaA06RGA/BnaC07RGA*, *BnaA06RGA/BnaA09GAI/BnaC09GAI*,

*BnaC07RGA/BnaC09GAI/BnaA09GAI*, *BnaA02RGL1/BnaC02RGL1*, *BnaA10RGL3/BnaC09RGL3*, and one tandem duplication *BnaA05RGL2/BnaA05RGL2-2* was determined (Fig. 5), which exhibits that during evolution segmental duplication events were the main reason for the divergence of the *DELLA* gene family in *B. napus*. In addition, comparative synteny of *DELLA* gene pairs between *B. napus*, *A. thaliana, B. rapa, B. oleracea,* and *B. nigra* was conducted (Fig. 6). The result shows that *BnaDELLAs* displayed the most collinearity relationship with *B. rapa*, *B. oleracea,* followed by *A. thaliana,* and *B. nigra.* A total of five, five, and 10 *BnaDELLAs* showed syntenic relationships with *B. rapa*, *B. oleracea*, and *B. nigra*, respectively (Table S2). However*,* five *AtDELLAs* show a collinearity relationship with 10 *BnaDELLAs*, which are more than one orthologous copy in the *B. napus* genome. For instance, *AtGAI* and *AtRGA* show syntenic relationships with *BnaA09GAI*, *BnaC09GAI*, *BnaA06RGA,* and *BnaC07RGA,* implying that *AtDELLAs* genes might contribute to the evolution of the *DELLA* gene family in *Brassicaceae* species. Moreover, we also evaluated the pressure of selective constraint on each pair of duplicated *BnaDELLAs* and calculated the nonsynonymous (Ka) and synonymous (Ks) ratio (Table S3, Figure S3). Our findings showed that all of the *BnaDELLA* pairs had the Ka/Ks ratio lower than 1, indicating that the *BnaDELLA* gene family experienced strong purifying selective pressure.

### Prediction of the bna-microRNAs putative targets site*s*

The regulatory purpose of *DELLAs* and their interacting targets have been characterized widely in various plant species; however, a possible underlying post-transcriptional modification of *DELLAs* in response to environmental stresses is still unclear [50–52]. It has been reported that miRNAs play a significant role in transcriptional and post-transcriptional levels to modulate gene expression under stresses [28, 53]. To identify miRNAs interaction with *BnaDELLAs* isoforms, we obtained the bna-miRNAs data from *B. napus* comprehensive study to predict the targeted *BnaDELLAs* sites. We found that 10 *BnaDELLAs* from *B. napus* targets for 18 conserved *B. napus* miRNAs. These miRNAs lengths reached from 1–24 base pairs, with 11 nt being the most frequent in all *BnaDELLAs* (Table 2). Target prediction analysis shows that *BnaDELLAs BnaC07RGA* and *BnaA09GAI* are targeted by two well-known miRNAs, bna-miR6029 and bna-miR6031, respectively. Among the other bna-miRNAs identified in our study, bna-miR2111a, bna-miR166a are found to be involved in targeting the *BnaRGL1*. In contrast, bna-miR172b targets *BnaA02RGL1* and *BnaA05RGL2*. Additionally, bna-miR390a and bna-miR168a are found to target *BnaRGL3*. Based on this analysis, we perceived that bna-miRNAs potentially target *B. napus,* both A and C genome, to regulate *BnaDELLAs* expression under constant stresses to stabilize plant growth and defense tradeoff.

**Table 2:** bna-miRNA targets BnaDELLA genes

miRNA_Acc	Gene symbol	Target_Acc	Target start	Target end	miRNA aligned fragment	Alignment length	e-value
bna-miR6029	*BnaC07RGA*	*BnaC07G0269400ZS*	590	610	UGGGGUUGUGAUUUCAGGCUU	21	5
bna-miR6031	*BnaA09GAI*	*BnaA09G0218400ZS*	1667	1690	AAGAGGUUCGGAGCGGUUUGAAGC	24	5
bna-miR6036	*BnaA09GAI*	*BnaA09G0218400ZS*	1581	1591	AUAGUACUAGUACUUGCAUGAUCA	11	5.5
	*BnaC09GAI*	*BnaC09G0254100ZS*	1551	1561		11	5.4
	*BnaA06RGA*	*BnaA06G0409200ZS*	1563	1573		11	5.4
	*BnaC07RGA*	*BnaC07G0269400ZS*	566	578		13	0.35
bna-miR6028	*BnaC09GAI*	*BnaC09G0254100ZS*	1635	1645	UGGAGAGUAAGGACAUUCAGA	11	5.4
	*BnaA06RGA*	*BnaA06G0409200ZS*	1647	1657		11	5.4
	*BnaC07RGA*	*BnaC07G0269400ZS*	1659	1669		11	5.4
bna-miR390a	*BnaA06RGA*	*BnaA06G0409200ZS*	275	285	AAGCUCAGGAGGGAUAGCGCC	11	5.4
bna-miR171f	*BnaA06RGA*	*BnaA06G0409200ZS*	888	898	UGAUUGAGCCGCGCCAAUAUC	11	5.4
bna-miR156b	*BnaC07RGA*	*BnaC07G0269400ZS*	108	123	UUGACAGAAGAUAGAGAGCAC	16	1.4
bna-miR168b	*BnaC07RGA*	*BnaC07G0269400ZS*	1079	1089	UCGCUUGGUGCAGGUCGAGAA	11	5.4
bna-miR160a	*BnaC07RGA*	*BnaC07G0269400ZS*	1530	1540	UGCCUGGCUCCCUGUAUGCCA	11	5.4
bna-miR2111a	*BnaC02RGL1*	*BnaC02G0205300ZS*	1375	1391	GUCCUCGGGAUGCGGAUUACC	17	0.31
bna-miR172b	*BnaC02RGL1*	*BnaC02G0205300ZS*	1038	1048	GGAAUCUUGAUGAUGCUGCAU	11	4.8
bna-miR166e	*BnaC02RGL1*	*BnaC02G0205300ZS*	612	623	UCGGACCAGGCUUCAUUCCCC	12	1.2
bna-miR166a	*BnaA02RGL1*	*BnaA02G0160500ZS*	1187	1198	UCGGACCAGGCUUCAUUCCCC	12	1.2
bna-miR6030	*BnaC02RGL1*	*BnaC02G0205300ZS*	736	746	UCCACCCAUACCAUACAGACCC	11	4.8
bna-miR2111c	*BnaC02RGL1*	*BnaC02G0205300ZS*	1288	1298	UAAUCUGCAUCCUGGGGUUUA	11	4.8
bna-miR172b	*BnaA02RGL1*	*BnaA02G0160500ZS*	1041	1051	GGAAUCUUGAUGAUGCUGCAU	11	4.8
	*BnaA05RGL2*	*BnaA05G0486300ZS*	745	755		11	5.1
bna-miR390a	*BnaA10RGL3*	*BnaA10G0194400ZS*	1442	1452	AAGCUCAGGAGGGAUAGCGCC	11	4.9
bna-miR168a	*BnaC09RGL3*	*BnaC09G0489900ZS*	404	414	AAGCUCAGGAGGGAUAGCGCC	11	4.9

### *cis*-element analysis in promoter regions of *BnaDELLAs* and their distribution

**Fig. 7:**
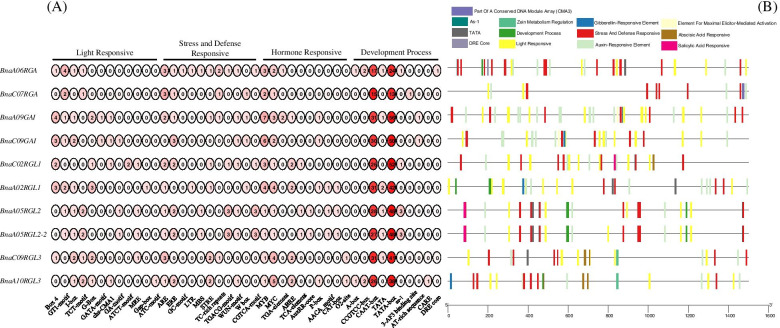
*cis*-acting element prediction in the *BnaDELLAs*. **A** The values in the circle indicated the count of *cis*-acting element in the promoter of *BnaDELLAs*; **B** The different colored block lines represent the different types and positions of *cis*-acting elements in each *BnaDELLA* gene

Physiological and molecular studies on *DELLAs* suggested their role in multiple hormonal signaling pathways by interacting with a wide variety of transcriptional regulators and transcriptional factors. However, the molecular mechanism of interaction and regulation of *DELLA* genes are quite unclear. To gain more insights into the potential function and regulatory mechanism of the *BnaDELLAs*, we analyzed the *cis*-regulatory elements in the 1500 bp upstream promoter region of the *BnaDELLAs* by using the plantCARE database and divided them into four categories (Fig. 7A). We found that the individual *DELLA* gene in *B. napus* contains multiple *cis*-acting elements (Table S4). Nearly all of the *BnaDELLAs* promoters have CAAT-box, TATA-box, light, stress, hormone, and development-related responsive *cis*-elements. However, the distribution and numbers varied significantly between the *BnaDELLA*s (Fig. 7B). In detail, *BnaA09GAI*, *BnaA02RGL1* has a higher number of light-responsive and hormone-responsive elements. In contrast, *BnaA06RGA*, *BnaA05RGL2,* and *BnaA10RGL3* carried a higher number of stress-responsive and development-related *cis*-elements, respectively. However, some of the *cis* core elements were only found in some *BnaDELLAs*. For example, GC-motif (enhancer-like element involved in anoxic specific inducibility), DRE-core (*cis*-acting regulatory element regulate cold stress, induce dehydration), and 3-AF binding site (part of a conserved DNA module array CMA3) were found in *BnaA06RGA* and *BnaC07RGA*. Similarly, GATA-motif (*cis*-acting regulatory element involved in light-responsive floral, hypocotyl, and seed development), AT-Rich sequence (*cis*-element for maximal elicitor-mediated activation) were present in *BnaCO9GAI* and *BnaA09GAI*. ATCT-motif (Part of a conserved DNA module involved in light responsiveness), Gap-Box (*cis*-acting element related to light-responsive GapA gene) was present in *BnaC02RGL1* and *BnaA02RGL1*, respectively. Moreover, AuxRR-Core (*cis*-acting regulatory element involved in auxin responsiveness) was only found in *BnaA05RGL2* and *BnaA05RGL2-2*. In contrast, O2-site (*cis*-regulatory element involved in zein metabolism regulation) was absent in all *BnaDELLAs* except *BnaC09RGL3* and *BnaA10RGL3* (Fig. 7A). These results showed that the *BnaDELLA* gene family contains a wide variety of stress and defense-related *cis*-elements compared to development, light, and hormone-responsive *cis*-elements, suggesting the *BnaDELLAs* diverse function in response to various biotic and abiotic stresses.

### Transcriptomic and qRT-PCR analysis of *BnaDELLAs* in different tissues

**Fig. 8:**
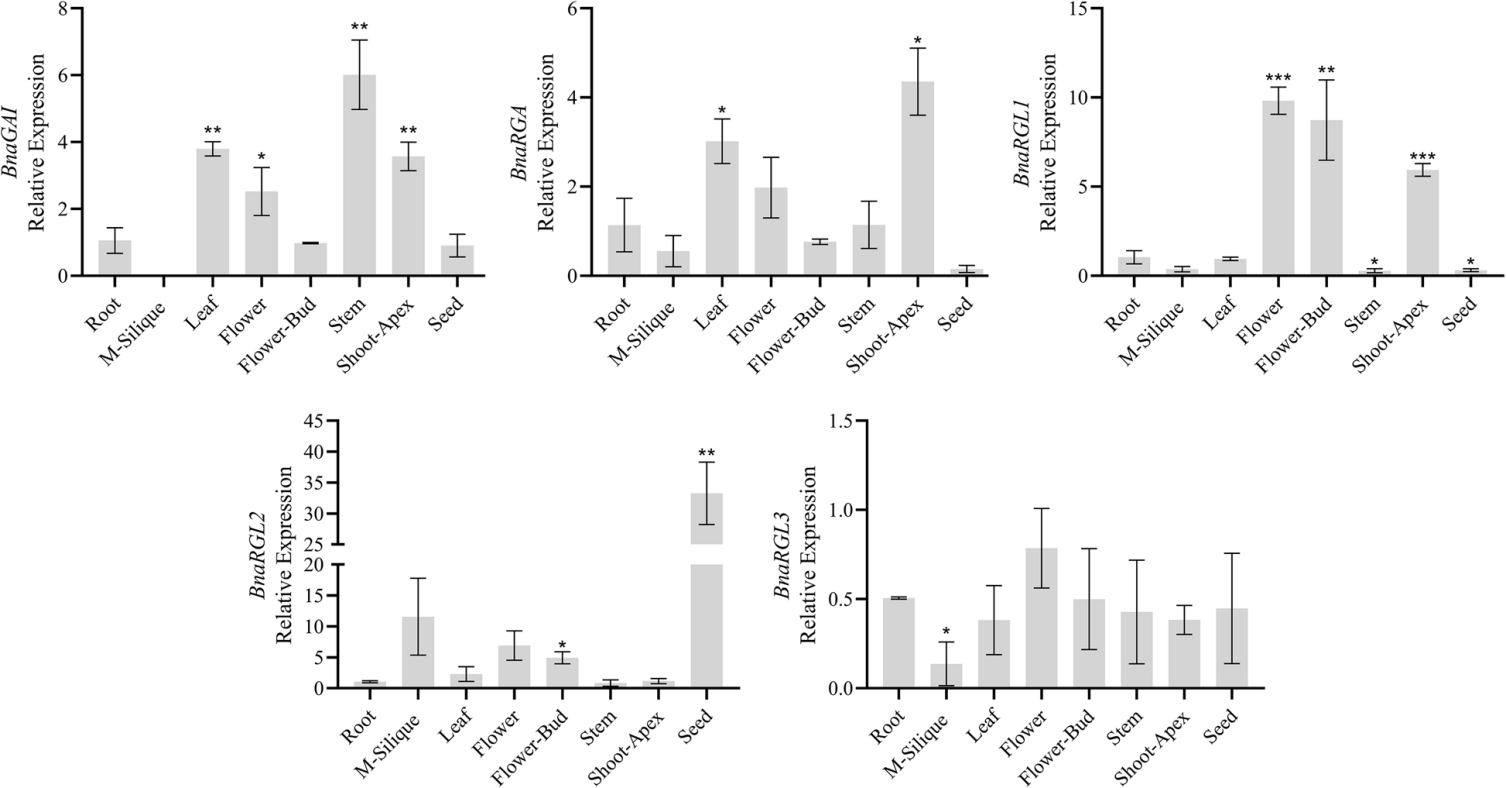
qRT-PCR analysis of the selected *BnaDELLAs* expression in different organs. The relative abundance of the selected *BnaDELLAs* was normalized with respect to the reference gene (*Actin*). The x-axis corresponds to different organs. Values on the y axis are denoted as the mean ± SD of three biological replicates (listed in Table S5.2). Asterias on vertical bar shows significant difference at * *P* < 0.05, ** *P* < 0.01, *** *P* < 0.001

The *BnaDELLA* gene family transcriptomic expression data from the roots, cotyledon, leaf, sepal, petal, filament, pollen, bud, middle stem, lower stem, upper stem, vegetative rosette, silique, silique wall, and seed of the *B. napus* cultivar ZS11, were obtained from the BnTIR database http://yanglab.hzau.edu.cn/BnTIR. The extracted data normalized by log2 fold change and heatmap was generated. As shown in Figure S4, the expression patterns of the 10 *BnaDELLAs* were different among roots, cotyledon, leaf, sepal, petal, filament, pollen, bud, middle stem, lower stem, upper stem, vegetative rosette, silique, silique wall, and seed, which points out that the additional copies of the homologs *BnaDELLAs* show variations in expression during seed germination to reproductive development. This can provide important insights into these genes distinct roles in *B. napus*. To better understand the expression pattern of the *BnaDELLAs*, we performed qRT-PCR in eight primary tissues (root, mature-silique, leaf, flower, flower-bud, stem, shoot-apex, seed) of *B. napus* cultivar ZS11. We found a strong correlation between the transcriptomic and our qRT-PCR results (Fig. 8). On the whole, *BnaGAI* and *BnaRGA* are highly expressed in the stem and shoot-apex, while *BnaRGL1* and *BnaRGL2* were mainly expressed in the floral organs and seed, respectively. Conversely, in our qRT-PCR analysis, *BnaRGL3* shows minimal expression in any tissues. However, combined with transcriptomic data analysis, *BnaRGL3* expression was highly observed in the silique. The contradiction between the qRT-PCR and transcriptomic data, especially in the *BnaRGL3* expression, might be due to the harvesting of silique at six and 28 days after flowering, which show the complex variation of the *BnaDELLAs* from seed germination to vegetative and reproductive development. This result indicates the unique expression patterns of the *BnaDELLAs* at multiple plant tissues, which might play an indispensable role in regulating gibberellins and other phytohormones signals to mediate plant growth and survival tradeoff under constant stress conditions.

### Expression analysis of *BnaDELLAs* under different stress

**Fig. 9:**
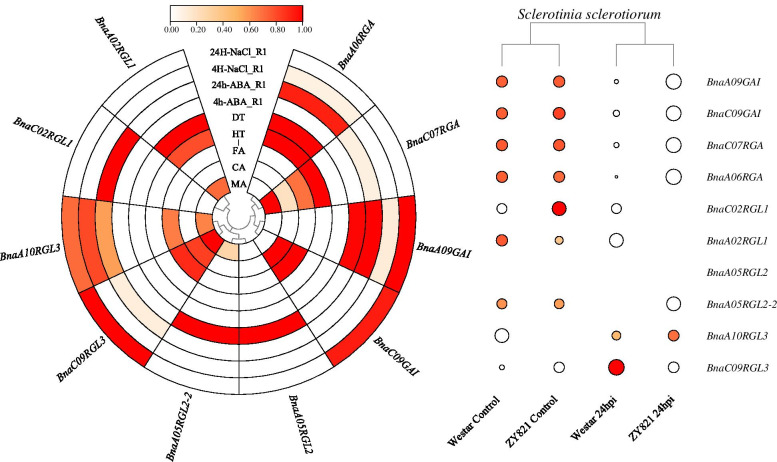
Heatmap of the expression profile of *BnaDELLAs* under different abiotic and biotic conditions including, MA (Cold shock at chilling 4 °C and freezing − 4 °C temperatures), CA (4 degree Celsius 12 h following cold acclimation 14 days 4 degree Celsius), FA (-4 degree Celsius 12 h following cold acclimation 14 days 4 degree Celsius), DT (Drought treated), HT (Heat treatment), ABA, salinity, and *Sclerotinia sclerotiorum.* The color scale reflects the data of the expression being processed with normalization of log2 (listed in Table S7), and different colors denote different expression levels

To further explore and gain more insights into possible *BnaDELLAs* function under biotic and abiotic stresses. We studied the pre-published RNA-seq data to detect the genes expression patterns under different stress conditions, such as MA (Cold shock at chilling 4 °C and freezing − 4 °C temperatures), CA (4 degree Celsius 12 h following cold acclimation 14 days 4 degree Celsius), FA (4 degree Celsius 12 h following cold acclimation 14 days 4 degree Celsius), DT (Drought treated), HT (Heat treatment), ABA (Abscisic acid), salinity, and *Sclerotinia sclerotiorum.* Overall, RNA-seq data analysis exhibits the *BnaDELLAs* expression patterns varied upon different stress treatments. For instance, *BnaRGL2* was up-regulated by all denoted stresses except in drought and salt (Fig. 9). Whereas, *BnaGAI* show putatively induced expression in response to MA, HT, DT, and salinity. In contrast, *BnaA10RGL3*, *BnaC09RGL3* almost exhibits reduced expression in response to heat, drought, ABA, and salt treatment. However, higher expression was observed during cold and *Sclerotinia sclerotiorum* treatment. Many previous studies on *AtDELLA* genes have provided evidence of their distinct and fundamental role in regulating plant physiology under abiotic and biotic stresses [33, 54–56], suggesting the strong relation of the *BnaDELLA* gene family in improving stress tolerance.

### Gene Ontology

In order to understand the functional regulatory mechanism of the *BnaDELLA* gene family, we used the *AtDELLA* orthologous pairs of the *A. thaliana* to performed GO enrichment analysis. Three common categories of GO terms were observed including, biological process (BP), cellular component (CC), and molecular function (MF). In the MF category, DELLA genes are highly enriched in binding (GO:0,003,700), (GO:0,005,515), and transcriptional regulation activity (GO:0,140,110). CC is enriched in the nucleus (GO:0,005,634), which exhibits that DELLAs are nuclear proteins. Similarly, most of the GO terms (GO:0,009,737, GO:0,009,739, GO:0,009,740, GO:0,009,753, GO:0,042,538, GO:0,009,863, GO:0,072,593, GO:0,009,651, GO:0,009,908, GO:2,000,033, GO:0,030,154, GO:0,010,187, GO:0,009,938, GO:0,006,355, GO:0,010,218, GO:0,009,723) were abundant in biological process, indicating a response to hormones and stresses (Figure S5, Table S8). This GO enrichment results suggested that the *BnaDELLAs* play a pivotal role in regulating hormonal signaling in response to stresses, which is consistent with previous studies [9, 57–59].

## Discussion

In this study, 10 putative *BnaDELLAs* were identified from the *B. napus* genome and grouped into three subfamilies *BnaGAI*/*BnaRGA*, *BnaRGL1*, and *BnaRGL2/BnaRGL3* based on their homology. Systematic analyses such as phylogenetic relation, gene structure, motif composition, physicochemical properties, gene duplication, miRNA prediction, and *cis*-element analysis in the promoters were performed. Moreover, qRT-PCR and pre-published RNA-seq data were analyzed to disclose the expression profiling of the *BnaDELLAs*. These results provide valuable insight for further functional characterization of the *BnaDELLA* gene family, which could improve molecular breeding to accommodate rapeseed plants to the expected climate conditions.

DELLA proteins are well-known as negative coregulators that mediate crosstalk between GAs and various hormonal signals to maintain plant growth and survival tradeoff, responding to abiotic and biotic conditions [8, 10]. Previous reports on the seeded plant had identified one, two, and five *DELLA* genes in *Oryza sativa* [25], *Pisum sativum* [27], and *A. thaliana*, respectively. Cloning and modulating DELLA proteins in these plants resulted in increased harvest index, seed quality, tillering, flower timing, and stress tolerance. For example, overaccumulation of the DELLA protein enhances the submergence tolerance [60], salt stress [61], and shade avoidance [62, 63], which significantly improves plant fitness. In contrast, reduced DELLA protein expression decreases tillering [64, 65] and seed dormancy [66], thus increasing seed weight and germination. In this study, a total of 10 *BnaDELLAs* have been identified in *B. napus*, which means that the individual *AtDELLA* have multiple homologs in *B. napus*. Rapeseed is an allotetraploid (AACC) crop that originated from the hybridization of two diploid progenitors *B. rapa* (AA) and *B. oleracea* (CC) [67]. Chromosomal mapping indicated that five and four *BnaDELLAs* are located on the proximal or the distal ends of AA and CC subgenome, respectively (Fig. 4), which exhibits that homologs of *BnaDELLAs* might play a similar role in biological function as both ancestral species.

DELLA gene family contain two highly conserved N-terminal DELLA and C-terminal GRAS domain in various plant species. In this study, it was found that the *BnaDELLA* gene family shared similar types of conserved domains. However, motif numbers and their composition between *BnaDELLAs* are unevenly distributed, indicating the domain shuffling in the protein structure of the *BnaDELLAs*, which may suggest functional diversification of the *BnaDELLA* gene family.

*DELLA* gene family in *A. thaliana*, *B. napus, B. rapa, B. oleracea,* and *B. juncea* shows a significant gene structure containing a single exon and does not have any introns. It has been shown that the genes with no or fewer introns expressed rapidly in response to biotic and abiotic stresses [68, 69]. Compared with transcriptomic data used in this study, we detected the distinct expression patterns of the intronless *BnaDELLAs* in response to cold, drought, heat, *Sclerotinia sclerotiorum,* salinity, and ABA treatments, suggesting the strong relation of *BnaDELLAs* to biotic and abiotic stresses (Fig. 9, Table S7). Moreover, exon composition exhibited the higher evolutionary conservation of *DELLA* genes among *Brassicaceae* species (Fig. 2).

DELLA proteins are well described as master repressors of GAs signaling to modulate plant physiology [70, 71]. GAs derepress DELLA repression through several positive regulators, including GA receptors GA-INSENSITIVE DWARF 1 (GID1), SPINDLY (SPY), and F-box protein (SLY1, SNE) under natural environment to stimulate plant growth [44]. However, several studies have illustrated that the *DELLAs* stability can be regulated through GAs dependent and independent proteolysis [72, 73]. A recent study has hypothesized that the rice microRNA (OsmiR396) putatively regulates the rice DELLA gene *SLR1*, targeting GA-responsive growth-regulating factors (GRFs) to inhibit growth promotion in rice [74]. In this study, a total of 18 bna-miRNAs were predicted in targeting the *BnaDELLAs* (Table 2). In which, *BnaC07RGA* and *BnaA09GAI* are putatively regulated by the two known miRNAs bna-miR6029 and bna-miR6031, respectively. In compliance with this, a recent study has shown that the increased expression of the bna-miR6029 regulates fatty acid biosynthesis to mediate seed development in response to environmental challenges [75]. Thus, we speculate that the *BnaDELLAs* were the most likely targeted genes by the predicted bna-miRNAs to mediate plant growth and survival tradeoff under constant exogenous or endogenous stimuli. However, further investigation is needed to elucidate the miRNA process with *BnaDELLA* genes.

This study also discovers diverse *cis*-elements in *BnaDELLAs* promoter, including light-responsive, hormones responsive, and stress-related elements (Fig. 7), but their distribution is uneven. For instance, *BnaA02RGL1*, *BnaC02RGL1,* and *BnaA09RGL3*, *BnaC09RGL3* had two ABREs in their promoter regions, while *BnaA05RGL2* and *BnaA05RGL2*-2 had no ABREs, although they were considered to induced ABA response differently. Additionally, *BnaA05RGL2* and *BnaA05RGL2*-2 had one MBS *cis*-element in their promoter regions. Intriguingly, the *BnaRGL2* gene relative expression was not observed in the drought treatment (Fig. 9). Thus, these findings indicate the presence of unidentified *cis*-elements and signify that the expression of *BnaDELLAs* might be regulated through post-transcriptional modification [50, 52], which provides the clue for gene expression studies under different biotic and abiotic stresses. Researches on *A. thaliana* have identified five *AtDELLA* genes GA-Insensitive (*GAI*), Repressor of ga1-3 (*RGA*), RGA-Like1 (*RGL1*), (*RGL2*), (*RGL3*). Cloning and sequencing of these *AtDELLA* genes reported the distinct and overlapping role in regulating GAs stimulated plant growth. For instance, *AtGAI* and *AtRGA* control hypocotyl cell division and floral induction [29, 30, 76]. *AtRGL1* and *AtRGL2* are involved in modulating leaf senescence, male sterility, and seed germination [32, 33]. While *AtRGL3* has been reported to contribute plant defense in response to biotic stresses [10, 35, 77]. Consistent with this, our gene expression profiling and pre-published RNA-Seq data analysis (Table S5, Table S7) putatively indicate the distinct expression patterns of the *BnaDELLA* gene family in response to biotic and abiotic stresses. For instance, *BnaRGL2* shows higher expression in all tested stresses except in drought, salinity, and *Sclerotinia sclerotiorum* (Fig. 9). Whereas, *BnaGAI* is expressed in stems and shows a response to MA, HT, DT, and salinity. In contrast, *BnaRGL3* almost exhibits reduced expression in response to heat, drought, and ABA treatment. However, induced expression was observed during cold and salt treatment. These findings are consistent with studies that have also been found on their homologs in *A. thaliana* [78, 79]. Moreover, previous studies also confirmed the increased expression of the *AtRGL3* in response to the plant defense [10, 35, 77]. Combined with transcriptomic data used in this study, we observed the increased expression of the *BnaA09RGL3* and *BnaC09RGL3* in 24 h of *Sclerotinia sclerotiorum* infection (Fig. 9), suggesting the *BnaRGL3* vital role in mediating *B. napus* survival under constant stress condition. Furthermore, *BnaRGL2* homolog in *A. thaliana AtRGL2* is indicated as an essential component to positively regulate ABA responses to promote seed dormancy [80–82]. In our qRT-PCR and RNA-seq analysis, we found that *BnaRGL2* was mainly expressed in the seeds and putatively showed induced expression after 4 h of ABA treatment but eventually reduced after 24 h of ABA treatment. However, further experimental studies are required to gain more insights into the *BnaDELLAs* in the ABA signal transduction pathway. In contrast, during salt stress, the transcripts of the *BnaA09GAI*, *BnaC09GAI,* and *BnaCO9RGL3* were up-regulated, whereas the rest of the *BnaDELLAs* were down-regulated (Fig. 9), suggesting the importance of *BnaGAI* in susceptibility to severe salt stress. Importantly the link of *AtGAI* with salt stress has been identified, which confirmed the enhanced salt tolerance by restraining the plant growth [83, 84].

Our study provides functional diversification and comprehensive knowledge of the *BnaDELLA* gene family *in B. napus.* However, further experimental studies are needed to better understand the distinct roles of the *BnaDELLAs* under biotic and abiotic stress conditions, which will help consolidate our understanding of plant ontogenesis and enhance agronomic techniques to improve *B. napus* yield.

## Conclusions

A significant role of DELLA proteins is to mediate GAs and almost all phytohormones signaling pathways to maintain a dilemma between plant defense and growth under constant stresses. In our study, we identified and characterized the *BnaDELLA* gene family in *B. napus*. A total of 10 *BnaDELLAs* have been identified in the *B. napus* genome and classified into three groups. All of the *BnaDELLAs* are closely related to the *A. thaliana* five *DELLA* genes, suggesting a comparable function and gene structure. The motifs composition within the same subfamily is uneven; however, individual *BnaDELLA* gene contains 12 highly conserved motifs, encoding the DELLA and GRAS domains. Phylogenetic and syntenic study of the *DELLA* genes between *B. napus* and its ancestral species provides helpful hints or evolutionary features of the *BnaDELLAs*. Moreover, miRNAs targets, *cis*-acting elements, and transcriptional regulation of the *BnaDELLA* gene family were also predicted. Overall, these results provide valuable clues into the evolutionary relationship and potential functions of the *BnaDELLAs*, which will be helpful for further genetic manipulation toward developing *B. napus* variants with enhanced tolerance to environmental fluctuation.

## Methods

### Identification and protein sequence analysis of *BnaDELLAs*

In order to search the *DELLA* gene family in *B. napus*, the peptide sequence of the five *DELLA* genes from *A. thaliana* genome database (http://www.arabidopsis.org/) with corresponding Gene ID (*At1G14920.1, At2G01570.1, At1G66350.1, At3G03450.1, At5G17490.1*) were retrieved and used as queries to perform BLAST P search in *B. napus* Genome browser (BnPIR, http://cbi.hzau.edu.cn/bnapus), and (GENOSCOPE, https://www.genoscope.cns.fr/brassicanapus/). Those from *B. oleracea*, *B. rapa*, *B. juncea*, and *B. nigra* were downloaded from *Brassica* Database (BRAD, http://brassicadb.cn/#/). The sequences with 80% similarity were selected, and incorrectly or repeated sequences were manually re-annotated for DELLA domain analysis in the scan ScanProsite (https://prosite.expasy.org) and InterProScan (https://www.ebi.ac.uk/interpro/search/sequence/). The protein sequences were then used to calculate the isoelectric point (pI), molecular weight (MW), and the number of amino acids by the ProtParm tool (http://web.expasy.org/). Furthermore, prediction of subcellular location pattern of each *BnaDELLA* was carried out using the web-server Plant-mPLoc (http://www.csbio.sjtu.edu.cn/bioinf/plant-multi/) [85], and ProtComp v.9.0 in softberry (http://linux1.softberry.com/).

### Phylogenetic and gene structure assessment of the DELLA in *B. napus, A. thaliana, B. rapa, B. oleracea, B, juncea, B. nigra*

Putative peptide sequences from the six *Brassicaceae* species *B. napus*, *A. thaliana*, *B. rapa*, *B. oleracea*, *B. juncea,* and *B. nigra* aligned using the MUSCLE (https://www.ebi.ac.uk/Tools/msa/muscle) with default parameters. Aligned sequences were then used to construct the evolutionary tree with the MEGA 10.2 software by the neighbor-joining (NJ) method [86]. The authenticity of the tree was tested by performing 1000 bootstrap replications. The phylogenetic tree Newick format was then uploaded to the iTOL web server (http://itol.embl.de/) for better visualization. Furthermore, genomic and coding sequences of the *B. napus, B.oleracea, B.rapa, B. juncea,* and *A. thaliana DELLA* genes were rendered in Gene Structure Display Server (GSDS2.0) (http://gsds.cbi.pku.edu.cn) to predict gene structure and exon/intron location.

### Sequence alignment and evaluation of *BnaDELLAs* motifs

To classify the DELLAs characteristic domains in the *B. napus*, we have aligned the 38 *DELLAs* codding sequence from *B. napus*, *A. thaliana*, *B. rapa*, *B. oleracea*, *B. juncea,* and *B. nigra* by using the Muscle option in the MEGA 10 with default parameters. Furthermore, Motif Elicitation version 5.1.1 (MEME http://meme-suite.org/tools/meme) was used to identify the conserved motifs in the *BnaDELLAs* with the maximum motif search set to 20, and other parameters are set to default. Any repetitions were considered motifs sites that spread throughout the sequence [87]. Further annotation of the identified motifs was implemented by the InterProScan (InterPro ebi.ac.uk). The conserved motifs were visualized by using the TBtools software [88]. Additionally, the secondary structure of the BnaDELLA proteins is carried by PSIPRED (http://bioinf.cs.ucl.ac.uk/PSIPRED).

### Chromosome location, collinearity analysis, and site-specific selection assessment and testing

*BnaDELLAs* detailed chromosome location was acquired from the GFF genome file downloaded from *B. napus* genomic database (BnPIR, http://cbi.hzau.edu.cn/bnapus), and mapped the predicted location on the chromosome by using the TBtools software with red-colored gene names indicated as relative position. Gene duplication events were identified by aligning the *BnaDELLAs* sequences using BLASTP and MCScanX to characterize the *BnaDELLAs* into a tandem and segmental duplication [89]. Furthermore, the syntenic map of *DELLAs* orthologous among *B. napus*, *A. thaliana, B.rapa, B. oleracea,* and *B. nigra* were obtained by the custom phyton script*.* For examining the site-specific selection, a Bayesian inference approach Selecton Server (http://selecton.tau.ac.il/ [90] was used to predict the positive and purifying selection. Besides this, we also calculated the synonymous (*Ks*) and nonsynonymous mutation (*Ka*) at each codon by *KaKs*_Calculator 2.0 [91]. In addition, *BnaDELLA* gene pairs divergence time was presumed using the formula T = Ks/2r with r (1.5 × 10^–8^) representing neutral substitution per site per year [92].

### miRNA target prediction and *cis*-acting elements regulatory analysis

To validate the interactions between miRNA and their targets. We obtained the *B. napus* stem-loop and mature miRNA sequences from the PNRD (http://structuralbiology.cau.edu.cn/PNRD/index.php) [93] and miRbase (http://www.mirbase.org/) database. The Plant small RNA Target analysis server psiRNATarget [94] with default parameters was used to predict the bna-miRNAs target genes in the *BnaDELLA* gene family. For *cis*-element analysis, 1500 bp upstream promoter sequence from the translation start site of the *BnaDELLAs* were inspected in the plantCARE database (http://bioinformatics.psb.ugent.be/webtools/plantcare/html/) [95], and distribution of the *cis*-acting elements visualized by TBtools software [88].

### Plant material RNA extraction and qRT-PCR

The seeds of *B. napus* cultivar ‘ZS11’ was donated by professor Liu Shengyi of Oil Crops Research Institute,

Chinese Academy of Agricultural Sciences, Wuhan. *B. napus* was grown in the greenhouse of Institute of Life sciences Jiangsu University under the following conditions 20 ± 5°C, 16 h light /8 h dark at a light intensity of 50 µmol/m2/s and 70% relative humidity. Tissues from roots, mature-silique, leaves, flowers, flower-bud, stems, shoot-apex, and seeds were collected from adult plants and immediately frozen in liquid nitrogen and stored at -80^ο^C for RNA extraction. The total RNA was extracted using Trizol (Invitrogen, Carlsbad, CA) and treated with RNase-free DNaseI (Invitrogen, Carlsbad, CA). Total RNA was then employed to produce cDNA with HiScript III-RT SuperMix for qPCR (Vazyme, China) according to the manufacturer's instructions. Real-time fluorescence quantitative analysis (qRT-PCR) was performed by Thermo Fisher Scientific QuantStudio 5 Real-Time PCR system with three independent replicates. The *B. napus Actin* gene (GenBank ID: XM_013858992) was used as an internal control. The 2^−∆∆^Ct method was implemented to measure the relative gene expression level of *BnaDELLAs*. The relative expression of the *BnaDELLAs* in root was used as control, and a t-test was implemented to measure the significant difference among tissues, and the results were visualized using GraphPad Prism8.0 software [96]. All of the gene-specific primers used in this study were designed by the Beacon primer design program (Primer Biosoft International, Palo Alto, CA) and listed in (Table S6).

### Gene ontology and expression pattern analysis of *BnaDELLAs*

The *BnaDELLAs* functional properties were analyzed using the online web server DAVID (https://david.ncifcrf.gov/) and panther (http://go.pantherdb.org/webservices/go/overrep.jsp) to conduct Gene Ontology enrichment analysis. The predicted GO terms were annotated using the TBtools software. In addition, expression profiles of *BnaDELLAs* under heat, drought, cold, ABA induce, salt and *Sclerotinia sclerotiorum* stress condition were obtained from the pre-published transcriptomic data sets (SRP277041), (SRP190170) [97], (CRA001775) [98], and (SRP075294) [99]. The differential expression analysis was performed using the DSEeq2 package in R. The predicted values were normalized by log2 fold change, and heatmap was generated via TBtools.

## Supplementary Information

Additional file 1: **Figure S1**: Alignment of BnaDELLA protein family.

Additional file 2: **Figure S2**: Schematic diagram of BnaDELLA proteins motifs logo.

Additional file 3: **Figure S3**: Site-specific selection assessment of BnaDELLAs.

Additional file 4: **Figure S4**: BnaDELLAs expression at different development stages in different organs by transcriptomic analysis.

Additional file 5: **Figure S5**: Gene ontology (GO) analysis.

Additional file 6: **Table S1**. Percent sequence homology comparison between B. napus and A. thaliana. **Table S2**. BnaDELLAs synteny relationship among A. thaliana, B. rapa, B. oleracea, B. nigra. **Table S3**. Ks/Ka values of BnaDELLAs. **Table S4**. cis-elements of BnaDELLAs. **Table S5.1**. BnaDELLAs expression at different development stages in different organs by transcriptomic analysis. **Table S5.2**: BnaDELLAs expression in different organs by qRT-PCR. **Table S6**: Primers used for Real-time PCR analysis. **Table S7.** Expression profiling of BnaDELLAs under different stresses. **Table S8**. GO enrichment analysis. **Table S9**. List and sequence of the 10 BnaDELLAs identified in this study.

## Data Availability

The genome, protein, and genome transfer format (GTF) file of *Arabidopsis thaliana, Brassica napus, Brassica rapa, Brassica oleracea,* and *Brassica nigra* was downloaded from the Ensemble FTP download (http://plants.ensembl.org/info/data/ftp/index.html). The RNA seq data under salinity and ABA treatment were available under the project ID: CRA001775 (https://bigd.big.ac.cn/). The supporting transcriptomic datasets have been deposited in the NCBI Sequence Read Archive (SRA, https://www.ncbi.nlm.nih.gov/sra) repository under accession numbers: SRP277041, SRP190170, and SRP075294. All data that support this study are included within the article and its additional files.

## Abbreviations

*B. napus*: *Brassica napus*
*A. thaliana*: *Arabidopsis thaliana*
*B. rapa*: *Brassica rapa*
*B. oleracea*: *Brassica oleracea*
*B. nigra*: *Brassica nigra*
*B. juncea*: *Brassica juncea*
*AtDELLA*: *Arabidopsis thaliana DELLA*
*BnaDELLA*: *Brassica napus DELLA*
GAs: Gibberellins
GO: Gene ontology
BLASTP: Basic local alignment search tool-protein
MW: Molecular weight
MEME: Motif elicitation
bna-miRNAs: *B**rassica napus* microRNAs
ZS11: Zhongshuang 11
GID1: Gibberellin insensitive Dwarf 1
GRAVY: Grand average of hydrophobicity
pI: Isoelectric point
ABA: Abscisic acid
NaCl: Sodium chloride
GSDS: Gene structure display server
CDS: Coding sequence
