# Supplementary material for: Genome-wide analysis and functional characterization of the DELLA gene family associated with stress tolerance in B. napus
Source: BMC Plant Biol. 2021 Jun 22;21:286. doi: 10.1186/s12870-021-03054-x (PMC8220683; doi:10.1186/s12870-021-03054-x)
Supplement: Supplementary file 1 — Figure S1: Alignment of BnaDELLA protein family. [file 12870_2021_3054_MOESM1_ESM.pdf]

Figure. S1

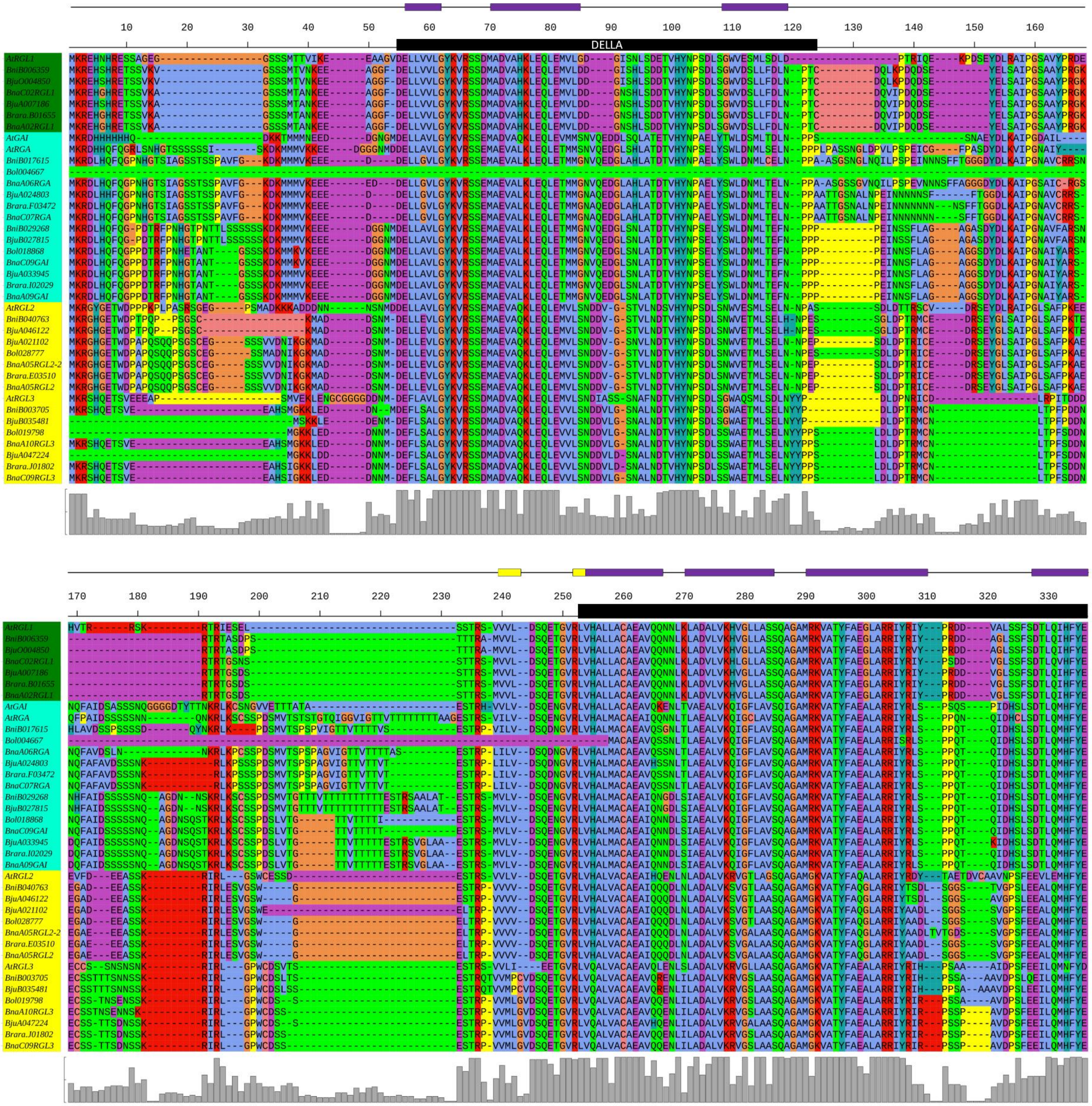
DELLA
AtRGL1
BniB006359
BjuO004850
BnaC02RGL1
BjuA007186
Brara.B01655
BnaA02RGL1
AtGAI
AtRGA
BniB017615
Bol004667
BnaA06RGA
BjuA024803
Brara.F03472
BnaC07RGA
BniB029268
BjuB027815
Bol018868
BnaC09GAI
BjuA033945
Brara.I02029
BnaA09GAI
AtRGL2
BniB040763
BjuA046122
BjuA021102
Bol028777
BnaA05RGL2-2
Brara.E03510
BnaA05RGL2
AtRGL3
BniB003705
BjuB035481
Bol019798
BnaA10RGL3
BjuA047224
Brara.J01802
BnaC09RGL3

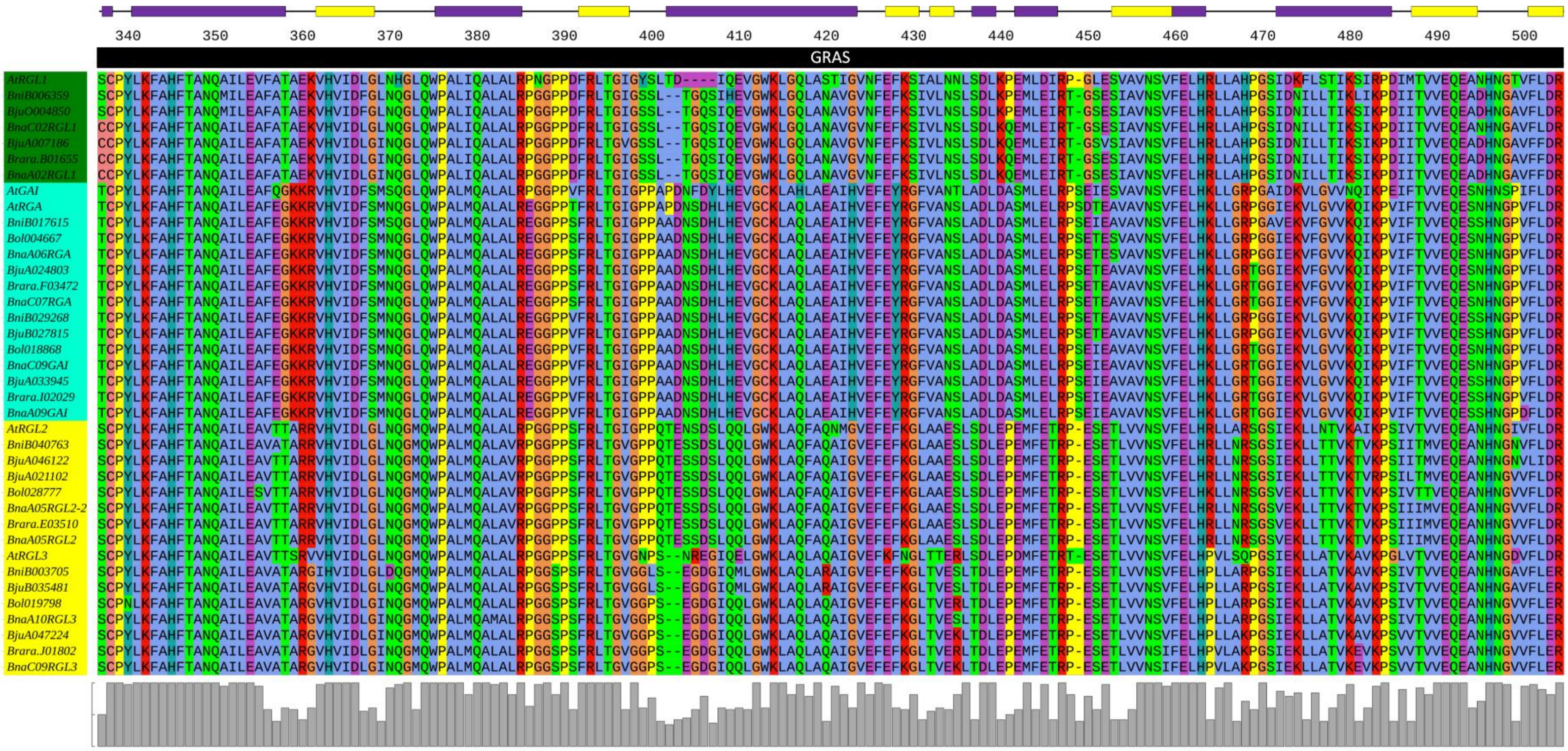
340
350
360
370
380
390
400
410
420
430
440
450
460
470
480
490
500
GRAS
AtRGL1
BniB006359
BjuO004850
BnaC02RGL1
BjuA007186
Brara.B01655
BnaA02RGL1
AtGAI
AtRGA
BniB017615
Bol004667
BnaA06RGA
BjuA024803
Brara.F03472
BnaC07RGA
BniB029268
BjuB027815
Bol018868
BnaC09GAI
BjuA033945
Brara.I02029
BnaA09GAI
AtRGL2
BniB040763
BjuA046122
BjuA021102
Bol028777
BnaA05RGL2-2
Brara.E03510
BnaA05RGL2
AtRGL3
BniB003705
BjuB035481
Bol019798
BnaA10RGL3
BjuA047224
Brara.J01802
BnaC09RGL3

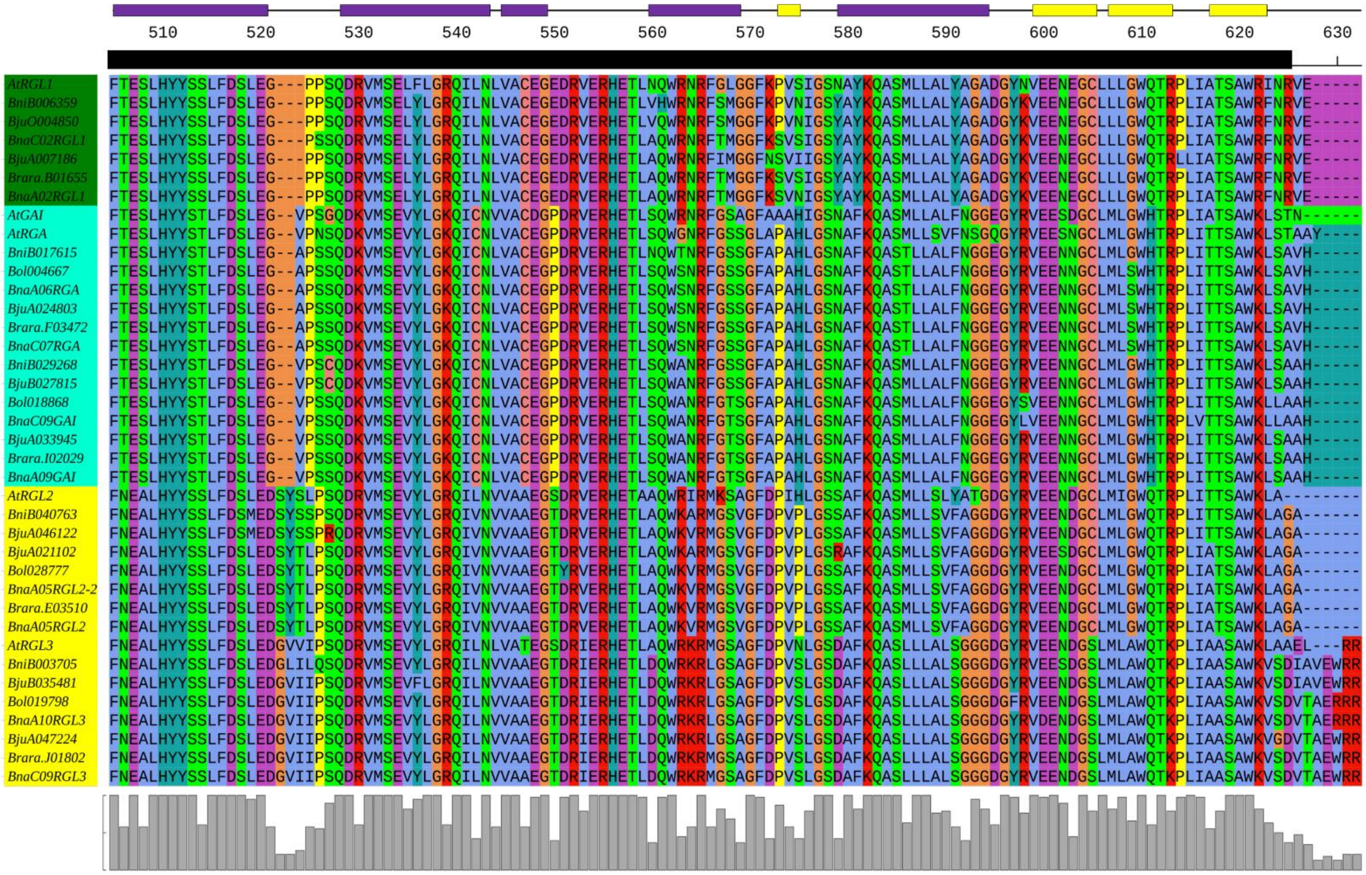
510
520
530
540
550
560
570
580
590
600
610
620
630
AtRGL1
BniB006359
BjuO004850
BnaC02RGL1
BjuA007186
Brara.B01655
BnaA02RGL1
AtGAI
AtRGA
BniB017615
Bol004667
BnaA06RGA
BjuA024803
Brara.F03472
BnaC07RGA
BniB029268
BjuB027815
Bol018868
BnaC09GAI
BjuA033945
Brara.I02029
BnaA09GAI
AtRGL2
BniB040763
BjuA046122
BjuA021102
Bol028777
BnaA05RGL2-2
Brara.E03510
BnaA05RGL2
AtRGL3
BniB003705
BjuB035481
Bol019798
BnaA10RGL3
BjuA047224
Brara.J01802
BnaC09RGL3

Figure. S1 Multiple sequence alignment of 38 members of the DELLA protein family. The groups of the DELLA proteins are indicated as different colors. The multiple alignment result clearly shows the highly conserved DELLA and GRAS domain among six *Brassicaceae* species. Absolutely conserved residues are highlighted in different colors. The predicted secondary structure α-helices in magenta, coils in black lines, β sheets in yellow were represented above the alignment.
