# Supplementary figures and images for: Genome-wide analysis and functional characterization of the DELLA gene family associated with stress tolerance in B. napus
Source: BMC Plant Biol. 2021 Jun 22;21:286. doi: 10.1186/s12870-021-03054-x (PMC8220683; doi:10.1186/s12870-021-03054-x)

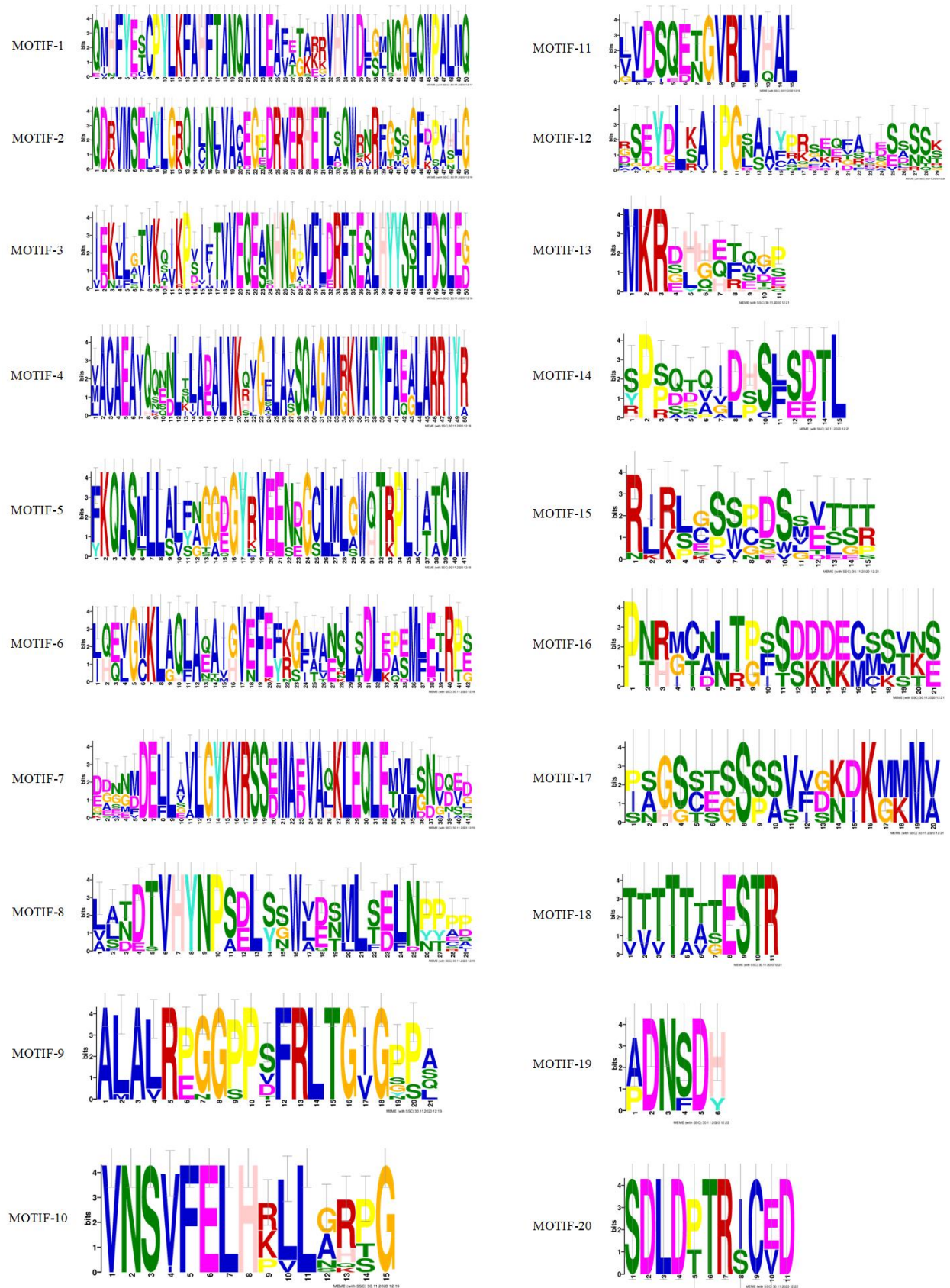

**Figure. S2** Schematic diagram of all *BnDELLAs* motifs logos

Supplement: Supplementary file 2 — Figure S2: Schematic diagram of BnaDELLA proteins motifs logo. [file 12870_2021_3054_MOESM2_ESM.pdf]
