# Supplementary material for: Genome-wide analysis and functional characterization of the DELLA gene family associated with stress tolerance in B. napus
Source: BMC Plant Biol. 2021 Jun 22;21:286. doi: 10.1186/s12870-021-03054-x (PMC8220683; doi:10.1186/s12870-021-03054-x)
Supplement: Supplementary file 3 — Figure S3: Site-specific selection assessment of BnaDELLAs. [file 12870_2021_3054_MOESM3_ESM.pdf]

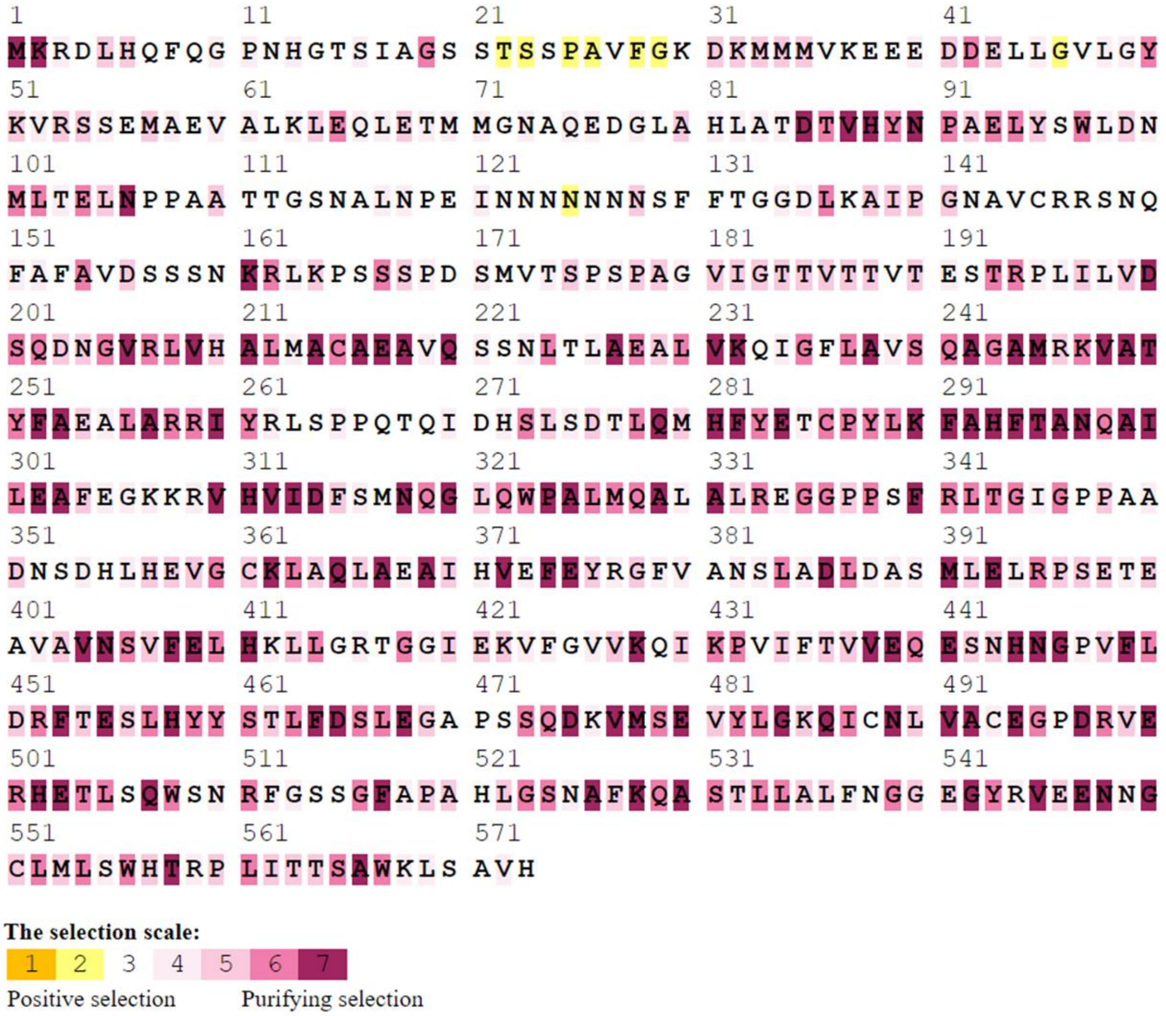

Figure. S3 site-specific selection assessment of *BnDELLAs*
