# Supplementary material for: Genome-wide analysis and functional characterization of the DELLA gene family associated with stress tolerance in B. napus
Source: BMC Plant Biol. 2021 Jun 22;21:286. doi: 10.1186/s12870-021-03054-x (PMC8220683; doi:10.1186/s12870-021-03054-x)
Supplement: Supplementary file 4 — Figure S4: BnaDELLAs expression at different development stages in different organs by transcriptomic analysis. [file 12870_2021_3054_MOESM4_ESM.pdf]

Figure S4

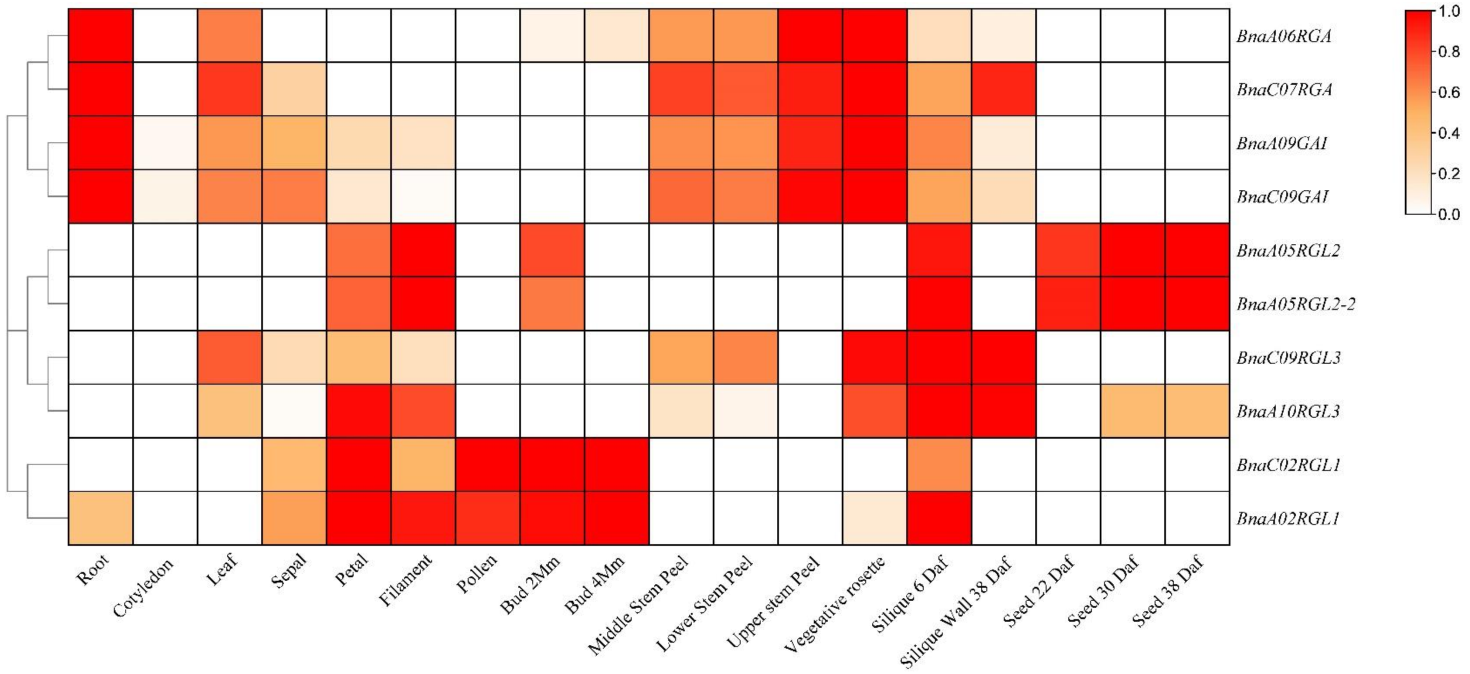

Figure. S4 *BnaDELLAs* expression at different development stages in different organs by transcriptomic analysis. Expression data were analyzed with log2 normalization. Relative expression levels from high (dark-colored) to low (light color) are represented by the color scale bar (listed in Table S5.1).
