# Supplementary material for: Genome-wide analysis and functional characterization of the DELLA gene family associated with stress tolerance in B. napus
Source: BMC Plant Biol. 2021 Jun 22;21:286. doi: 10.1186/s12870-021-03054-x (PMC8220683; doi:10.1186/s12870-021-03054-x)
Supplement: Supplementary file 5 — Figure S5: Gene ontology (GO) analysis. [file 12870_2021_3054_MOESM5_ESM.pdf]

Figure S5

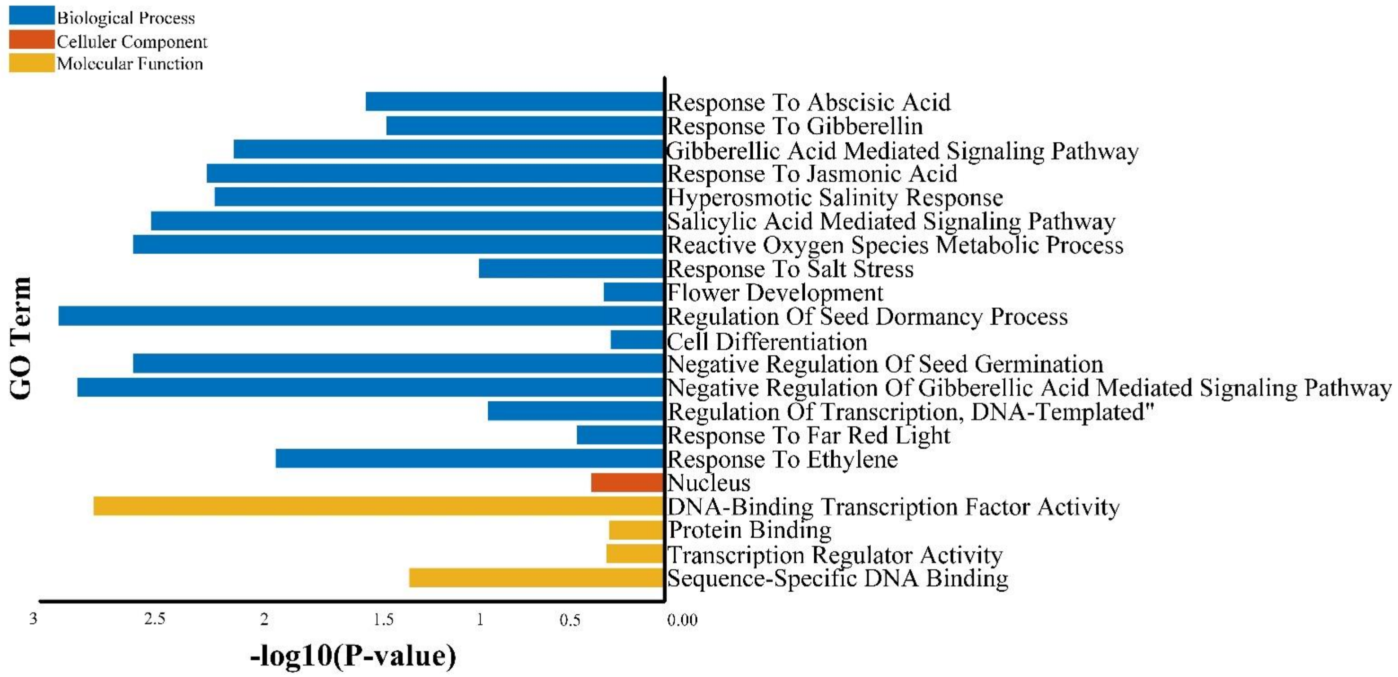

Figure. S5 Gene ontology (GO) analysis. Three categories represent as designated colors, Blue (Biological Process), Red (Cellular Component), Orange (Molecular Function), were used to perform annotation on BnaDELLA proteins.
